# Work-family boundary management profiles and well-being at work: A study with militaries on a humanitarian aid mission

**DOI:** 10.1080/08995605.2023.2195793

**Published:** 2023-04-06

**Authors:** Maria José Chambel, Vânia Sofia Carvalho, Francisco Gomes, Carolina Rodrigues-Silveira

**Affiliations:** Faculdade de Psicologia, Universidade de Lisboa, Lisboa, Portugal

**Keywords:** Boundary management, exhaustion, engagement, person-centered approach, boundary segmentation, boundary preferences

## Abstract

This study aims to contribute to an analysis of the well-being of military personnel who are deployed on humanitarian aid missions, taking their work-family (personal life) boundary management into consideration by analyzing the relationship between their preferences and enacted boundaries and military personnel’ well-being. Specifically, this study analyzed the boundary fit approach, positing that it is the adjustment between individuals’ preferences and enacted boundaries that influences their well-being. Using a sample of 327 military personnel, boundary management profiles were performed, considering the fit between their segmentation preferences and enactment. Furthermore, the relationship between these profiles and the military personnel’ well-being was established. The results indicated that misfit profiles were found where the soldiers enacted less segmentation than desired or, on the contrary, more integration than desired, and a profile with a fit between the work-family segmentation they desired and enacted. The military personnel in the fit profile had significantly higher levels of well-being (i.e.,less exhaustion and more work engagement) than those in the misfit profile, who enacted less segmentation than desired. The findings have implications for the design of boundary management literature and future military missions.

**What is the public significance of this article?—**This study suggests that military boundary management on a non-combat mission may be challenging once it affects their well-being. The boundary management is more challenging when there is a misfit between preferences for segmentation/integration and enacted boundaries, with a negative impact on well-being. On the contrary, a fit between preferences for segmentation/integration and enacted boundary management segmentation/integration has positive effects on military well-being. Overall, it reinforces the importance of boundary management coherence.

## Introduction

The work-family relationship (personal life) has emerged as a prevalent research theme over recent decades (Eby et al., 2005; Liu et al., 2019) given the importance of the relationship between these domains in explaining individual well-being (Michel et al., 2011). In fact, the boundaries established between the two domains have been found to have relevant repercussions for the balance obtained and, consequently, for how individuals feel (Allen et al., 2014; Clark, 2000). On one extreme, rigid and rather impermeable boundaries are established that create a *segmentation* of the two domains, keeping them separate, such as the case of a professional with two cell phones, one professional the other personal, who only keeps the former switched on when working and turns it off as soon as his work schedule has ended to turn on his personal device. Conversely, on the opposite extreme, flexible and permeable boundaries are established that create an *integration* of the two domains, where there is mutual interference, such as the case of a professional who has only one cell phone that is constantly switched on and may be used during working hours both for personal and family calls and to resolve work-related issues after his work schedule has ended (Bulger et al., 2007). These boundaries may be of a physical nature, establishing a difference between the workplace and the family (personal) life location, of a temporal nature, defining the moment when each role should be performed, namely working hours and leisure time, and of a psychological nature, establishing the appropriate emotions, behaviors and thoughts for each domain (Allen et al., 2014).

In the case of military personnel deployed on missions, such as those providing humanitarian aid, establishing these boundaries may be viewed as a challenge since they are away from their families and live and work in the same location. As there is no physical boundary, it might be difficult for them to create a temporal and psychological boundary. Furthermore, the permeability and flexibility of their family (personal) domain in relation to their professional domain may give rise to an invasion of the latter, fostering the feeling that they live and breathe their work to the exclusion of all else (Ashforth et al., 2000; Clark, 2000). However, the boundaries of these domains are not entirely defined by contextual conditions. They are also dependent on the actions taken by individuals to manage these boundaries which, in turn, are also reliant upon both their preferences and the policies and actions of the organization in supporting them in this management (Rothbard & Ollier-Malaterre, 2016; Kossek & Lautsch, 2012; Nippert-Eng, 1996). For example, during a mission, a soldier may wish to keep these two domains *segmented* and reserve a period each day after work hours to contact his/her family, however this is only possible if his/her supervisors do not delegate him/her a task to be carried out at the same time. Moreover, as stated by Ammons (2013), in order to gain a better understanding of boundary management, it is necessary to analyze whether the individual’s boundary preferences and boundary created (i.e., enacted) are aligned, since this analysis sheds light upon the influence of boundary management on an individual’s health and well-being.

Thus, the aim of this study is to contribute to an analysis of the well-being of military personnel who are deployed on humanitarian aid missions, taking their work-family (personal life) boundary management into consideration by analyzing the relationship between their preferences and enacted boundaries. Firstly, one of its main contributions is the consideration that work-family (personal life) boundary management may explain the well-being of military personnel during a mission. Although deployment to non-combat missions is increasingly more frequent in military life and is known to involve uncommon psychological challenges that may compromise the well-being of the military personnel who participate in these missions (Campbell & Nobel, 2009; King et al., 1995), studies with humanitarian aid military personnel are almost non-existant (Britt & Adler, 1999 may be seen as an exception). Since these professionals are away from their families and living and working in the same location, the establishment and maintenance of boundaries between the professional and family (personal) domains is likely to be a difficult task, thus compromising the balance between these two domains and, consequently, the well-being of the military personnel (Ashforth et al., 2000). Secondly, and in line with the person–organization fit literature (Kristof-Brown et al., 2005), this study analyzed the boundary fit approach, positing that it is the adjustment between individuals’ preferences and enacted boundaries that influences their well-being. Ammons (2013) empirically tested this assumption in a qualitative study, however in this study a person-centered methodology is used, namely profile anaysis. This methodology has the advantage of providing knowledge on the patterns of individuals who share the same way of managing the work-family boundary. To our knowledge, only three studies have used this person-centered approach to analyze boundary management profiles, namely those of Kossek et al. (2012), Moazami-Goodarzi et al. (2015), and Kinnunen et al. (2016). However, these studies focus more on individuals’ control over boundaries, their segmentation/integration tendencies and the behaviors from one domain interrupting the role in the other domain. Unlike this study, they do not focus on preferences and enacted boundaries in boundary management.

In short, this study stands apart from others in the field, due to its innovative approach of taking Ammons (2013) idea of congruence/incongruence between preferences and enacted boundaries as its starting point, but by means of a more robust data analysis methodology, and by using a sample of participants experiencing a situation that fosters the interference of work in family life, thus hindering work-family segmentation.

## Theoretical framework

### Boundary management profiles

Work-family (private life) boundary management may be defined on a continuum between *segmentation*, the extreme on which professional life and personal life are separate, and *integration*, the other extreme on which they are both mixed and convergent (Kreiner, 2006; Nippert-Eng, 1996). This management is bidirectional and both the relationship of work with family and of family with work may take on multiple configurations. For example, some individuals may keep the family domain separate from the work domain (family-work segmentation) but allow work to interfere in the family domain (work-family integration) or vice versa, while others keep the work domain separate from the family (work-family segmentation) but allow the family domain to interfere in their work (family-work integration; Ammons, 2013; Kossek et al., 2012).

In this type of management, it is possible to distinguish between the desire or preference and the actual boundary that is enacted. The former characterizes the individual’s preferences for the establishment of physical, temporal, cognitive and behavioral elements to define the boundary between the two domains, while the latter refers to the enacted boundary created to demarcate these domains (Ashforth et al., 2000; Kossek et al., 2005; Kreiner, 2006). Since the enacted boundary is dependent on the organization’s policies and practices, promoting different levels of segmentation between these domains (Kreiner, 2006; Olson-Buchanan & Boswell, 2006), there may be *fit* situations, where individuals are able to manage the boundary between these domains in accordance with their preferences, or *misfit* situations, where their management does not correspond to their preference. As found in the qualitative study of Ammons (2013), individuals have different enacted boundaries – the “protecting family*”*strategy, enacted by individuals who manage to achieve segmented management between the domains; the “above and beyond*”* strategy, adopted by those who integrate work into the family but segment work from the family; the opposite “enhancing family*”* strategy, enacted by individuals who keep work outside the family domain but integrate the family domain into work; and the “holistic boundaries*”* strategy, based on an integration of both domains. In each group, there are individuals who enact a strategy that *fits* their preferences, while there are also others who experience a *misfit.*

In the case of military personnel on a humanitarian aid mission despite living in the workplace where it is difficult to segment the professional and family domains, different boundary management profiles may be foreseen. Some military personnel will establish integration between the two domains in accordance with their preference, since by always being available to respond to mission-related demands, for example, they are able to establish contact with their families or engage in leisure activities when it is more suitable for the mission. Other military personnel, on the contrary, will manage to segment the professional and family domains according to their preference as, for instance, they establish schedules that separate the two domains and make contact with their families/friends during their free time which is respected by their supervisors and colleagues who do not make professional demands on them outside their work schedule. Others will experience a misfit situation between a strong preference to segment these two domains and that which is effectively enacted since, for example, they have to interrupt their leisure activities to respond to professional demands or as a result of their colleagues making constant references to mission occurrences during moments of leisure, thus not allowing them to maintain their desired distance from their professional activity. Others, conversely, may segment the boundaries of the two domains to a higher degree than is preferrable as the mission activities are so demanding that the military personnel are unable to even think about their families (personal life) during their accomplishment, and also any contact with their families can only be established during moments of leisure, despite their preference for greater flexibility and the possibility of sometimes interrupting their activity to contact their families.

### Boundary management profiles and well-being

Several studies have shown that the *segmentation* of family and professional domains leads to better outcomes and, conversely, the *integration* of these domains is conducive to poorer outcomes (Allen et al., 2014; Olson-Buchanan & Boswell, 2006; Park & Jex, 2011; Powell & Greenhaus, 2010). In fact, as far as stress repercussions are concerned, considering that stress is a result of resource loss, threat of resource loss and resource investment without replacement (Hobfoll, 2002), the integration of domains may be deemed a stressful situation. When there are permeable and flexible boundaries resulting from an invasion of one domain into the other, individuals invest resources in the performance of the role of the intrusive domain. This results in fewer resources for the performance of the role in the invaded domain, thus creating a stressful situation (Park & Jex, 2011). On the contrary, given that well-being results from resource acquisition ou maintenance (Hobfoll, 2002), when the domains are *segmented*, individuals have more resources (e.g., energy, time, availability) for the performance of their role in each domain, which fosters their well-being (Hobfoll, 2002). In the same vein, organizational contexts have been shown to foster workers’ domain segmentation (e.g., organizations do not allow their workers to prolong their work schedule and additionally establish prohibitive contact rules – telephone calls and e-mails – only outside working hours), thus protecting their resource loss, preventing the development of stress and promoting their well-being (Thompson & Prottas, 2006).

Boundary fit, namely the fit between the preferred and enacted boundary, may on the other hand be expected to influence individuals’ stress and well-being (Ammons, 2013). In this regard, the person–organization fit theory (P–O fit; Kristof-Brown et al., 2005) suggests that negative effects (e.g., high stress and low well-being) will result from a discrepancy between individuals’ needs (e.g., segmentation preference) and enactment (e.g., the segmentation obtained). When individuals prefer domain segmentation and this preference is supported in their work context by means of conditions and resources that allow them to manage these boundaries according to their preference, they experience less stress and enhanced well-being (Chen et al., 2009). However, when there is incongruence, and the conditions in the workplace do not allow workers to establish their desired domain segmentation, they experience resource loss, which, in turn, threatens their well-being (Kreiner, 2006).

In the case of military personnel on a military humanitarian aid mission, given the afore-mentioned blurred physical boundary between these two domains, it may be particularly difficult for them to segment their work and family life and, consequently, work will invade their family (personal) domain. It is difficult for military personnel with greater preference for segmentation to find a context that is congruent with their wishes. Therefore, worse consequences for these professionals may be expected when they experience less segmented (more integrated) situations between their professional life and family (personal) life, which is incongruent with their wishes (Paustian-Underdahl et al., 2016). On the other hand, these military personnel may benefit from a situation that allows them to segment these two domains since they will perceive they are being supported in the management of their professional and family (personal) lives in a manner that is in line with their preferences (Marescaux et al., 2020). However, the military personnel who prefer integration (or less segmentation) of the two domains will be less affected by the incongruence between their preferences and enacted boundaries. If they do not value clear boundaries between their professional and family (personal) lives, a context fostering the segmentation of these domains will not affect their stress or well-being (Foucreault et al., 2018).

Thus, it may be expected that the military personnel who prefer to segment the two domains and do not have this possibility in the context of the mission will experience more stress and less well-being. For example, the military personnel who like having their moments of leisure separated from their professional activity but are unable to manage this since they are constantly having to prolong their work schedule or respond to unexpected demands from their supervisors outside their work schedule, thus compromising the possibility of contact with their families, will experience a greater misfit between what they desire and what they effectively enact and will consequently experience more stress and less well-being. On the other hand, the military personnel who do not prefer segmentation between the professional and family domains will experience this frequent invasion of the professional domain in the family domain as being less *threatening* to their resources and this will consequently translate into fewer repercussions for their stress and well-being. However, if the military context provides these military personnel with the possibility of segmenting the two domains according to their preference, for example, by respecting schedules and not allowing supervisors or colleagues to make demands outside their work schedules, leaving them free to establish contact with their families or engage in sports activities during their free time, they may be expected to experience less stress and more well-being.

In this study, exhaustion and work engagement were analyzed as indicators of stress and well-being. Exhaustion, one of the dimensions of burnout, is characterized by a mental state of absence of physical, cognitive and physical resources to cope with the demands of the professional activity, pointing to stress in such contexts (Maslach, 1993). Work engagement, on the other hand, is considered a positive psychological state of well-being in the work context, resulting from constant resource acquisition (Bakker et al., 2014; Schaufeli & Bakker, 2004), thus enabling the individual to respond to the demands of this professional context (Hakanen et al., 2018).

Thus, the first aim was to analyze the boundary management profiles of military personnel on a humanitarian aid mission and the first hypothesis of the study is as follows:

**H1:** At least four profiles for military personnel’ boundary management are expected to be found: a fit profile where the military personnel prefer and enact a high level of segmentation between the professional domain and the family domain; a misfit profile where they would prefer to have greater segmentation between the two domains than that which they effectively enact; a misfit profile where the military personnel would prefer to have less segmentation (i.e., more integration) between the two domains than they effectively enact; a fit profile where they do not desire or enact segmentation (i.e., they enact integration) of the two domains.

The second aim was to analyze the relationship between the fit/misfit levels in the segmentation profiles with the stress (e.g., exhaustion) and well-being (e.g., work engagement) of the military personnel, according to which the following hypothesis is posited:

**H2:** The military personnel with a fit profile in which they prefer and enact high segmentation of their professional and personal (family) lives have the highest levels of well-being (i.e., lower exhaustion and higher work engagement) and, conversely, the military personnel with a misfit profile, in which they wish for greater segmentation of these domains than they enact, have the poorest levels of well-being (i.e., higher exhaustion and lower work engagement).

## Method

### Procedure and sample

This study was carried out with Brazilian military personnel who participated in Operation *Acolhida*, designed to provide emergency assistance to Venezuelan migrants and refugees as a result of the political and economic crisis in their homeland and the exodus of thousands of citizens. It took place on the border of Pacaraima-Romania, the main point of entry into Brazil from Venezuela. Following its approval by the Deontology Committee of the Scientific Council of the Faculty of Psychology of the University of Lisbon, the study and its aims were presented to the military personnel by a psychologist from CPAEx – Center for Applied Psychology of the Army (Brazil), and the anonymity of the participants was ensured. Once the informed consent had been signed, the data were collected by means of the military personnel’ responses to a questionnaire via the survey monkey platform approximately four months into the mission. At the end of the study, a report was drawn up with the global findings in simplified, non-technical language, and later delivered to the mission commander and the commander of CPAEx.

The sample consisted of 327 military personnel (72% of the military personnel on the mission), with the following characteristics: 290 (88.7%) were male and 37 (11.3%) were female; with a mean age of 32.3 (SD = 10.5) years; 144 (44%) were single, 164 (50.2%) were married or in a stable relationship and 19 (5.8%) were divorced or separated.

### Measures

#### Boundary management

The boundary management was measured throught scales of segmentation preference and enacted segmentation that we detailed below:

#### Segmentation preference

In order to measure segmentation preferences, three items of the scale of Kreiner (2006) were used to assess the work-family segmentation preference (e.g., “I prefer to think only about work when I’m working”) and three items of Methot et al. (2016) to assess the family-work segmentation preference (e.g., “I prefer to leave my personal life issues for my free time”). The participants were asked to assess each item in accordance with a five-point Likert type scale, ranging from *I strongly disagree* (1) to *I strongly agree* (5), where higher scores indicated greater preference for work-family segmentation (WFSP) and greater preference for family-work segmentation (FWSP). These scales had already been translated into Portuguese within the scope of the ISWAF project, and revised to adapt the language to the Brazilian Portuguese variant by two CPAEx psychologists. In this study, these two dimensions of the scale presented internal consistency indices of.70 and .73, respectively.

#### Enacted segmentation

In order to measure current segmentation behavior, six items of the scale of Chen et al. (2009) were used, three in enacted work-family segmentation (e.g., “I only think about work during the periods when I’m working”) and three in enacted family-work segmentation (e.g., “I leave my personal life for my periods of free time on the mission*”*). The participants were asked to assess each item in accordance with a five-point Likert type scale, ranging from *Never* (1) to *Very often* (5), where higher scores indicated higher enacted work-family segmentation (WFES) and higher enacted family-work segmentation (FWES). These scales had already been translated into Portuguese within the scope of the ISWAF project, and revised to adapt the language to the Brazilian Portuguese variant by two CPAEx psychologists. In this study, these two dimensions of the scale presented internal consistency indices of.72 and .75, respectively.

#### Well-being at work

We used the scales of exhaustion and work engagement to measure well-being at work.

#### Exhaustion

In order to assess exhaustion, the version validated for Brazil (Schuster et al., 2014) of the exhaustion subscale of the Maslach Burnout Inventory – General Scale (MBI-GS, Maslach et al., 1997) was used. The scale consists of five items (e.g., “ I feel tired when I get up in the morning and need to face another day of work”), with a seven-point Likert scale ranging from 1 (*Never*) to 7 (*Every day*). In this study, this scale presented an internal consistency index of .88.

#### Work engagement

To assess this positive psychological state in relation to work, the short version of Schaufeli et al. (2006) was used. The scale consists of nine items (e.g., *When I wake up in the morning, I feel good about going to work*), on a seven-point Likert scale, identical to the one used to measure exhaustion. This scale had already been used in studies with military personnel in Portugal (e.g., Carvalho & Chambel, 2018), and revised to adapt the language to the Brazilian Portuguese variant by two CPAEx psychologists. This scale presented an internal consistency index of .91.

### Data analysis

Four steps were followed to perform the analysis. In the first step, a Confirmatory Factor Analysis (CFA; Brown, 2015) with structural equation modeling methods was implemented with Mplus 7.2 (Muthén & Muthén, 2017). The recommendation of Podsakoff et al. (2003) to test the common error variance method was followed, applying Harman’s single-factor test, and performing a Confirmatory Factor Analysis of the studied variables.

The maximum likelihood estimation provides the well-known global fit statistics for structural equation modeling methods: the Comparative fit index (CFI; satisfactory values of 0.90 and above), the Tucker-Lewis index (TLI; satisfactory values of 0.90 and above), and the Root mean squared error of approximation (RMSEA; satisfactory value below 0.08) (van de Schoot et al., 2014) were used. To control for common method variance, our structural model was compared with a one-factor model (in which all items loaded onto a single latent variable).

In the second step, a Latent Profile Analysis (LPA) was conducted using the maximum likelihood estimator of MPLUS 7.2 to identify latent profiles. The optimal number of profiles was determined through an iterative process in which a two-profile model was estimated, and successive profiles were subsequently added (Nylund et al., 2007). Each model was evaluated using the following criteria: (a) the Bayesian information criterion (BIC), (b) entropy, and (c) the Lo-Mendell-Rubin adjusted likelihood radio test (LMR). The optimal solution should exhibit the following characteristics: (s) the lowest BIC (the BIC is used to select the model with the best fit and fewest parameters from a set of nonhierarchical models) and (b) the highest entropy value. Additionally, comparing neighboring LMR profiles against each other should yield significant p-values that indicate improvements in model fit as the number of profiles is successively increased.

## Results

### Measurement model

The goodness-of-fit index of the theoretical model (six-factor model, Preference for segmentation (Work to Family and Family to Work), Enacted Segmentation (Work to Family and Family to Work), Exhaustion, Work Engagement) presented an acceptable fit to the data: χ^2^ (333) = 827.70, *p* < .01, CFI = .90; TLI = .90; RMSEA = .07). A four-factor model was also performed where Preferred and Enacted scales were together in both work to family and family to work directions and Exhaustion and Work Engagement. The fit of this four-factor model was (χ^2^ (342) = 1660.61, *p* < .01, CFI = .74; TLI = .72; RMSEA = .07). The comparison with our theoretical measurement revealed that the measurement model fit best was (Δχ^2^ (17) = 2966.24, *p* < .01). A one-factor model was then performed, and the measurement model was observed to present a much better fit to the data than the one-factor model (χ^2^ (350) = 3193.94, *p* < .01, CFI = .45; TLI = .40; RMSEA = .16), which assumed that the data fit the theoretical model better than the one-factor model, Δχ^2^ (17) = 2966.24, *p* < .01 and confirmed the construct validity of the measurement model.

### Descriptive variables and correlations

By means of a correlations matrix (*r*), the variables were also observed to correlate with each other (*p* < .001). In particular, WFSP was positively related to FWES (*r* = .59, *p* < .001) as was FWSP to FWES (*r* = .59, *p* < .001) and WFSP (*r* = .44, *p* < .001). WFES was also found to be positively related to Work Engagement (*r* = .35, *p* < .001) which, in turn, was negatively related to Exhaustion (*r* = −.42, *p* < .001) (see Table 1).

**Table 1.** Means, standard deviation and correlations (r) among the variables under study (*n* = 327).

			*R*					
	Means	SD	1.	2.	3.	4.	5.	6.
1.FWPS	3.40	.95	-					
2.WFPS	3.41	.86	.44**	-				
3.WFES	3.39	.72	.20	.59**	-			
4. FWES	3.12	.86	.59**	.18**	−.01	-		
5.Work Eng.	5.36	1.04	−.23**	.10	.35**	−.13*	-	
6.Exhaustion	3.21	1.30	.31**	.04	−.22**	.15**	−.42**	-

*p < .05; **p < .01.

### Boundary management profiles

As illustrated in Table 2, of the various solutions tested, the five profile solution was the one that presented the best fit. Although its p value is higher than that of all the other solutions, this solution had the lowest BIC value and highest entropy value.

**Table 2.** Index fit for the four solutions estimated from the latent profile analysis for each sample.

Number of Profiles	BIC	Enthropy	LMR *p value*
2	3 209	.67	n.s.
3	3 129	.78	.01*
4	3 076	.80	.23*
5	3 019	.83	.05*
6	3 023	.81	n.s

*p < .001; n.s. = non significant.

**Figure 1.**
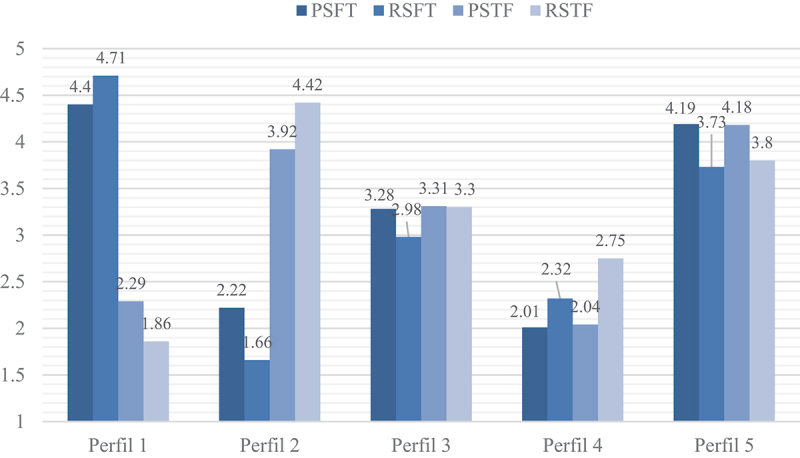
Mean of profiles for the five profile solution of segmentation preferences and enactment.

As may be seen in Figure 1, Profile 1 (segmenting family-work and integrating work-family) had a low percentage of participants (*n* = 16; 4.95%), characterized by a misfit between their preferences and enactment, higher family-work segmentation than desired, but also higher work-family integration than desired. Profile 2 (segmenting work-family and integrating family-work) had an equally low percentage of participants (*n* = 18; 5.57%), characterized by a misfit between their preferences and enactment, with higher family-work integration than desired, but also higher work-family segmentation than desired. Profile 3 (segmenting moderately in both directions) had the majority of participants (*n* = 168; 52.01%), characterized by a fit between their preferences and enactment in work-family segmentation, but by a misfit in family-work segmentation, since they enacted less segmentation than desired. Profile 4 (integrating in both directions) had a relatively low number of participants (*n* = 34; 10.53%), characterized by a misfit between their preferences and enacted boundaries, with less family-work and work-family integration than desired. Profile 5 (segmenting in both directions) was the second largest group of participants (*n* = 87; 26.93%), characterized by a misfit between their preferences and their enacted boundaries, with less family-work and work-family segmentation than desired.

As predicted in Hypothesis 1, several border management profiles were found between work and family, with misfit profiles and a fit profile between the preferences and enactment of the domains’ segmentation/integration, as also expected. As predicted, a misfit profile was found (Profile 5) where the military personnel wished to have more segmentation than they enacted, and another misfit profile (Profile 4) was also found where the military personnel enacted less integration than desired. However, as far as the other profiles are concerned, there are differences in terms of initial predictions: despite finding a fit profile (Profile 3) where the military personnel had the segmentation they desired, this fit only existed in the work-family segmentation, while in the family-work segmentation a misfit was observed since the military personnel had less segmentation than desired; although misfit profiles were found, where the participants enacted more segmentation than desired, the misfit only occurred in the family-work segmentation (Profile 1) and work-family segmentation (Profile 2), while in the opposite direction the misfit occurred due to the fact that the military personnel enacted more integration than desired. Thus, H1 was partially supported.

### FWSP, WFSP, WFES, and FWES profiles and well-being

In order to test Hypothesis 2, two ANOVAs were conducted, one in relation to exhaustion the other to work engagement (Table 3). According to Hypothesis 2, the military personnel with a fit profile where they desired and enacted a high level of segmentation between their professional and family (personal) life, were expected to have the highest levels of well-being (i.e., less exhaustion and more work engagement). Conversely, the military personnel with a misfit profile, where they enacted less segmentation between these two domains than desired, had the poorest levels of well-being (i.e., more exhaustion and less work engagement). As may be observed in the results, there is a significant difference between the profiles both in relation to exhaustion (F (323, 4) = 2.94, *p* < .05) and to work engagement (F (323, 4) = 2.66, *p* < .05). As expected, the military personnel in Profile 5 (a misfit where they enacted less segmentation than desired, in both directions) experienced higher levels of exhaustion and lower levels of work engagement than the military personnel in Profile 3 (fit between preference and enactment in work-family segmentation), as may be seen with the Post Hoc Bonferroni pairwise comparisons between the profiles in Table 3.

**Table 3.** Means (M) and Standard Deviation (SD) for strain and the five identified profiles.

	Profile 1 M (SD)	Profile 2 M (SD)	Profile 3 M (SD)	Profile 4 M (SD)	Profile 5 M (SD)	F value	Bonferroni pairwise comparisons between profiles^a^
Exhaustion	2.81 (1)	3.22 (1.4)	3.06 (1.3)	3.16 (1.5)	3.60 (1.2)	2.94*	5 < 3
Work Eng.	5.56 (1.1)	5.51 (.9)	5.48 (1)	5.34 (.9)	5.06 (1.1)	2.66*	3 < 5

*p < .01; ^a^the differences between and within profiles reported are significant at the level *p* < .05, the profiles represented are the only ones with significant differences; Work Eng. = Work Engagement.

## Discussion

Managing the boundaries between work and family may be a significant challenge for military personnel on a non-combat mission, as they are away from their families and live and work in the same location. Thus, the aim of this study with a sample of Brazilian military personnel on a humanitarian aid mission was not only to analyze their boundary management profiles, considering the fit between their segmentation preferences and enactment, but also the relationship between these profiles and the military personnel’ well-being. As expected, misfit profiles were found where the military personnel enacted less segmentation than desired or, on the contrary, more integration than desired, and a profile with a fit between the work-family segmentation they desired and enacted. Furthermore, as predicted, the military personnel in this fit profile had significantly higher levels of well-being (i.e., less exhaustion and more work engagement) than those in the misfit profile, who enacted less segmentation than desired.

In line with the qualitative study conducted by Ammons (2013), in this study the work- family boundary management profiles were analyzed, considering the fit between preference and enactment in terms of these domains’ segmentation. As expected, and in line with the findings of the afore-mentioned study, misfit profiles were observed where the preferences of the military personnel were not in line with their enacted segmentation/integration. It should be noted that among these misfit profiles, the profile with the highest number of military personnel was the one where, despite acknowledging the existence of segmentation between the domains, they wished for more segmentation. This result is in keeping with the conditions experienced by these military personnel since, owing to the fact that they are away from their families, the family domain is not likely to interfere significantly in their work (e.g., the soldier is unlikely to receive telephone calls from the family). However, as a result of this distance from the family, the military may have undesired thoughts and concerns related to this domain, even when working. Likewise, despite having to live and work in the same location, given the military context, a set of standards and rules are in place which allow for the domains to be segmented (e.g., strict working hours, leisure activities are encouraged). Even so, this may also give rise to undesired situations in which it is difficult for these military personnel to disconnect from their work (e.g., face-to-face contact solely with coworkers; the physical space fosters constant thinking about work issues). In fact, the conditions experienced by the military personnel also serve to explain why no fit profiles were found where the military personnel desired and enacted high levels of segmentation or integration, contrary to what was expected and to the findings of Ammons (2013). Only one fit profile was found, including over half of the participants, where the military personnel had a moderate preference and enacted moderate work-family segmentation. Being away from their families and living in army barracks are a part of military life, however the recruitment and training processes for participation in missions draw attention to these variables in order to prepare the military personnel for such conditions, thus making a fit between preference and enactment in moderate segmentation situations more likely than in extreme situations.

In line with the person–organization fit theory (P–O fit; Kristof-Brown et al., 2005), our hypothesis that the highest level of well-being would be observed in the fit profile and the lowest in the misfit profile, where the military personnel desired more segmentation than they enacted, was supported. Former studies (Chen et al., 2009; Kreiner, 2006; Liu et al., 2019) have already highlighted the positive effects of a fit between preference and the resources made available by organizations in work-family boundary management, however, this study has been pioneering both in its analysis of the boundary preferences and enacted boundary fit (Ammons, 2013), and in the use of Latent Profiles Analysis to test this fit. Firstly, it should be noted that this study sheds light upon the fact that beyond segmentation preferences (Park & Jex, 2011) or enacted segmentation (Thompson & Prottas, 2006), it is important to consider the fit between these two dimensions in order to understand the effect of segmentation on work-family boundary management. Indeed, in our misfit profile, where the military personnel desired more segmentation than they enacted, both the preferences and segmentation they had were higher than those of the military personnel in the fit profile. Nevertheless, it was in this latter profile that the best results were observed, namely less exhaustion and more work engagement. Secondly, the military personnel in the misfit profiles who had more segmentation (i.e. less integration) than desired were observed to be less affected by the incongruence between their preferences and the enacted boundaries, and there was no difference between their levels of exhaustion or work engagement. Thus, this study appears to support the assumption that not all misfit situations in work-family boundary management are translated into poorer outcomes, only those that prevent the preferred domain segmentation (Paustian-Underdahl et al., 2016). Conversely, in situations where the reality allows for more segmentation than desired, work-family boundary management is an easier task (Foucreault et al., 2018), involving less investment and resource loss and, consequently, no repercussions in terms of stress and well-being (Marescaux et al., 2020).

Finally, this study showed that the military personnel with higher well-being belonged to the profile with the work-family segmentation fit and the family-work segmentation misfit, since they desired more segmentation than they enacted. Possible explanations for this unexpected outcome may be related to the sample characteristics. As the sample consisted mainly of males in an institution with traditional values, namely in terms of gender roles (Eagly & Wood, 2016), it may be assumed that the professional role is more relevant than the family role and the work-family segmentation fit takes precedence over the work-family misfit (Beauregard, 2011). Moreover, as the military personnel are participating in a mission for which they volunteered, their identification with the career is likely to be enhanced (Lobel & Clair, 1992). These hypotheses require further research so that the possible effect and consequences both of gender and the relevance of professional and family roles on work-family (personal) boundary management may be analyzed.

### Limitations and suggestions for future research

This study presents some limitations which should be taken into consideration. The first lies in its temporal nature as only the preferences and enactment of the individuals’ management were analyzed at a single point in time, thus not allowing for a comparison of their stability across time, as conducted in the study of Ammons (2013), or the identification of antecedents and possible reasons behind the differences in the profiles.

Secondly, it should also be noted that this study used self-report questionnaires which carries the risk of common error variance, arising from individuals’ tendency to give socially desirable responses and their affective states (Podsakoff et al., 2003), even though anonymity was assured throughout the entire research process. Notwithstanding, the comparison of a one-factor model with the model of this study was carried out with a view to validating the fit of the latter. In fact, the Confirmatory Factor Analysis was also carried out to attenuate the risk associated with these questionnaires (Podsakoff et al., 2003).

Thirdly, some information, namely the length of these military personnel’ careers and their officer status, could not be collected to ensure anonymity, but may have influenced the segmentation enacted by the participants and consequently their inclusion in boundary profiles. Furthermore, segmentation behaviors may be affect by the presence of high demands or by specific deployment rules. As such, future studies should include these variables.

Finally, it should be noted that despite the representative sample of military personnel in this mission, the low number of participants called for a Latent Profile Analysis. Indeed, the number assigned to some profiles is low. Thus, we strongly encourage the replication of this study in similar operations with more participants.

Considering the above-mentioned limitations, future studies should include more information about the participants and not be limited solely to the identification of boundary management profiles at a single point in time. All the antecedents that contribute to this management and the consequent evolution of these profiles across time should also be analyzed. Following this line of thinking, future studies should avoid using a single source to obtain responses, and whenever possible opt for the highest number of sources.

Indeed, caution should be taken in future studies to analyze the segmentation-integration continuum in relation to the boundary management of the two domains (work and family), as highlighted by Bulger et al. (2007), since the real or desired segmentation/integration level may not be distributed in the same way in the two dimensions, as shown in this study. Moreover, as suggested by Ammons (2013), the research focus should shift to analyzing how the conditions of the different contexts influence boundary management preferences and enactment and the respective fit between them.

### Practical implications

Despite the above-mentioned limitations, this study represents a preliminary position within the scope of boundary management literature by assessing both the preferences and enactment experienced by the individuals and demonstrating the importance of their coherence.

It is necessary to highlight once again the sample of military personnel used in this study. Although attention has not been given to this type of population in boundary management literature, it has the potential to contribute to a better understanding of this subject, owing to the specific situation of these individuals. The characteristics that underlie this population force them to live in their workplace and to be away from their families for long periods of time, thus inevitably giving rise to blurred physical boundaries which, in turn, is positively related to both directions of the work-family conflict and more transitions between the work and family roles (Matthews et al., 2010). The prior preparation of these individuals for the circumstances they will experience is therefore crucial so that they can adapt their boundary management strategies and tactics “to create their ideal work-family segmentation or integration levels and styles” and minimize the work-family conflict (Kreiner et al., 2009).

This study, in line with several others (e.g., Chen et al., 2009; Kreiner, 2006; Liu et al., 2019), highlights the fundamental role of boundary management coherence for the well-being of military personnel. Thus, in the mission’s preparation, the army can diagnose the integration or segmentation preferences of the military personnel by means of questionnaires or interviews, for example. On the other hand, the supervisors on the mission should be made aware of the military personnel’ boundary management preferences so as to adapt the required measures in the theater of operations. During the mission, some of the army’s rules could be adapted to the military personnel’ preferences. For example, if a soldier prefers integration, contact with his relatives at uncommon hours could be facilitated or, on the contrary, if a soldier prefers segmentation, hours for family contact could be established in advance. Additionally, the spaces for leisure and work should be clearly defined with or without access restrictions during working hours, according to the military personnel’ preferences.

## Data Availability

The data that support the findings of this study are openly available in Mendeley Dataset at http://doi.org/10.17632/hmnrffbzdj.1.
